# Smart Eyeglasses: A Valid and Reliable Device to Assess Spatiotemporal Parameters during Gait

**DOI:** 10.3390/s22031196

**Published:** 2022-02-04

**Authors:** Justine Hellec, Frédéric Chorin, Andrea Castagnetti, Olivier Guérin, Serge S. Colson

**Affiliations:** 1 Université Côte d’Azur, LAMHESS, EUR HEALTHY, 06205 Nice, France; chorin.f@chu-nice.fr (F.C.); serge.colson@univ-cotedazur.fr (S.S.C.); 2 Ellcie Healthy, 06600 Antibes, France; 3 Université Côte d’Azur, CHU, Cimiez, Plateforme Fragilité, 06000 Nice, France; 4 Université Côte d’Azur, LEAT UMR 7248, 06410 Biot, France; andrea.castagnetti@univ-cotedazur.fr; 5 Université Côte d’Azur, IRCAN, 06107 Nice, France; guerin.o@chu-nice.fr

**Keywords:** wearable sensor, accelerometer, reproducibility, reliability, validity, spatiotemporal parameters

## Abstract

The study aims to determine the validity and reproducibility of step duration and step length parameters measured during walking in healthy participants using an accelerometer embedded in smart eyeglasses. Twenty young volunteers participated in two identical sessions comprising a 30 s gait assessment performed at three different treadmill speeds under two conditions (i.e., with and without a cervical collar). Spatiotemporal parameters (i.e., step duration and step length normalized by the lower limb length) were obtained with both the accelerometer embedded in smart eyeglasses and an optoelectronic system. The relative intra- and inter-session reliability of step duration and step length computed from the vertical acceleration data were excellent for all experimental conditions. An excellent absolute reliability was observed for the eyeglasses for all conditions and concurrent validity between systems was observed. An accelerometer incorporated in smart eyeglasses is accurate to measure step duration and step length during gait.

## 1. Introduction

Human gait is a complex functional ability that is acquired in childhood and progressively executed automatically in everyday life. People younger than 65 years old walk an average of 9800 steps per day, while this number reduces to 6500 steps for people older than 65 years [1]. As gait ability decreases with aging, gait analysis has been used as a biomarker for longevity [2], fall risk [3,4] and general health [5]. Numerous clinical tests exist (e.g., the 6 min walk test, the incremental shuttle walking test, the 10 m walk test), but during these clinical assessments, the spatiotemporal gait parameters are not routinely measured. However, the recording of spatiotemporal parameters is important to detect the risk of fall or frailty in elderly people [6], especially step duration [5,7] and step length [8,9]. Advanced age leads to a modification of the spatiotemporal parameters during walking [10]. Although the development of pressure walkways [11] or optical systems [12] permits the assessment of spatiotemporal gait parameters in or outside a laboratory environment, wireless and wearable technology has emerged recently.

Various wearable sensors, namely Inertial Measurement Units (IMUs), have been progressively implemented to assess gait [13,14], not only in laboratory settings [7], but also in ecological conditions [15,16,17,18]. The advantages of these sensors are numerous: low cost, small in size, lightweight, usable in everyday environments and do not require time-consuming analyses [4]. For these reasons, the number of studies conducted is increasing at an exponential rate, and recent reviews have summarized the relevance of IMUs for gait analysis in healthy adults [19], the elderly [4,20,21] and in various health-compromised populations [22,23,24,25]. While many gait analysis parameters have been considered, a recent systematic review and meta-analysis emphasized that, amongst others, spatiotemporal parameters, such as step duration and step length, measured with IMUs, have excellent and good validity, respectively [19,26,27].

During gait analysis, the IMUs were placed at the lower back (e.g., lumbar vertebrae, sacrum or pelvis), but other locations from the foot to the head were used (e.g., ankle, shank, knee, thigh, hip, wrist, elbow, upper back, sternum, shoulder [21,28]). The lower back is mainly used for convenience (i.e., “non-intrusive” long-term use) and to approximate the center of mass of the body. To discriminate fallers and non-fallers through the recording of spatiotemporal parameters during gait analysis, some authors placed an accelerometer at the level of the head [7,21]. This placement provided better kinematic information of the risk of fall compared to hip and trunk locations [29]. Considering that most elderly people wear glasses during activities of daily living, placing an IMU in such a non-intrusive wearable object of daily life could be a technological innovation enabling gait analysis. For example, in France, more than 90% of people aged 60 years old and over wear glasses and this percentage is about 40% for people aged as young as 20 to 50 years old [30]. Consequently, gait assessment with an IMU located at the head level seems promising for the quantification of spatiotemporal parameters in both laboratory and ecological conditions for clinical and research purposes. Although previously used for the assessment of the maximum vertical acceleration peak during the sit-to-stand movement [31], to our knowledge, no study has assessed spatiotemporal parameters of gait with an IMU embedded in glasses.

Therefore, the aim of this study is to evaluate the intra- and inter-session reliability, reproducibility, and concurrent validity of step duration and step length recorded with an IMU embedded in smart glasses in comparison with an optoelectronic system in young and healthy participants in a laboratory environment. 

## 2. Materials and Methods

### 2.1. Participants

Twenty healthy adults volunteered (10 men and 10 women) to participate in the study (age: 26.7 ± 4.6 years old; height: 173.5 ± 8.8 cm; body mass: 68.7 ± 15.4 kg; BMI: 22.6 ± 3.6 kg/m^2^; and lower limb length: 93.3 ± 4.5 cm; mean and standard deviation). For each participant, lower limb length was measured between the greater trochanter and the external malleolus for each side, and the average of both limbs was calculated. Inclusion criteria were being aged between 20 and 35 years old, being able to walk without a walking aid, having no optical correction, not having any known cognitive disorder, the absence of neuromuscular injury of the lower limbs in the last 12 months and voluntary participation. The experimental procedures were explained to the participants, and they gave their written informed consent before beginning the study. This study was conducted according to the Declaration of Helsinki revised in 2013 and was approved by the South Mediterranean Protection of Persons Ethics Committee (registration number: 2015-A01188-41).

### 2.2. Study Design

This single-group repeated-measures study was conducted in a gait analysis laboratory (Frailty Platform/Plateforme fragilité) at the University Hospital Center in Nice (France). Participants attended the laboratory on two separate identical testing sessions, separated by one week. During each testing session, participants walked on an instrumented treadmill (Bertec Corporation, Columbus, OH, USA) at three constant imposed speeds (i.e., low at 0.72 m/s, moderate at 0.90 m/s and high at 1.1 m/s). For each speed, participants had to walk in two experimental conditions (i.e., with or without the use of a cervical collar). Participants then performed six different experimental conditions during each session and the order of the conditions was randomized. For each experimental condition, participants walked at the selected speed for 30 s and were instructed to walk normally while looking straight ahead. The treadmill was switched on 10 s before the acquisition, to guarantee a constant speed during the 30 s of measurement. During all the testing sessions, the participants wore glasses without any optical correction.

### 2.3. Apparatus, Data Collection

**Figure 1:**
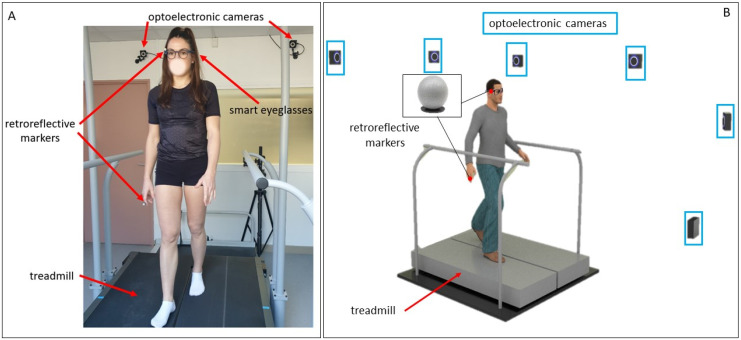
Participant walking on the treadmill with the equipment set up (**A**) and 3D schematic representation of the gait analysis laboratory (**B**).

**Figure 2:**
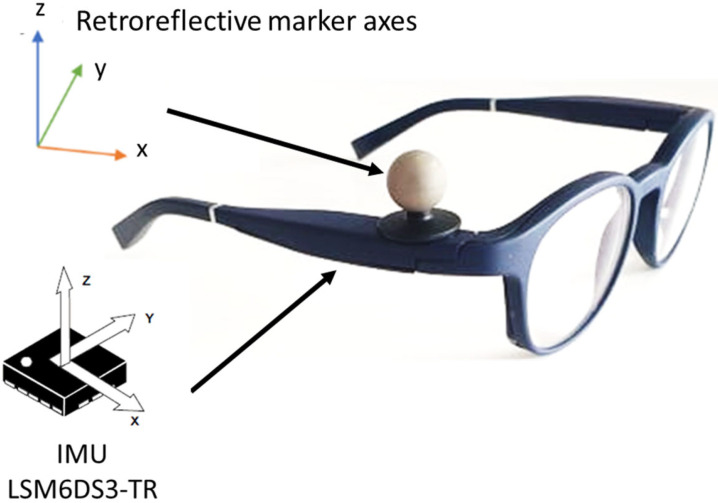
Position of the retroreflective marker on the right arm of the glasses and axes of the IMU and retroreflective marker.

The gait analysis laboratory was equipped with six optoelectronic cameras (OptiTrack system, Natural Point, Inc., Corvallis, OR, USA), which served as the reference measurement system. Along with a photograph of a participant during a testing session, a schematic representation of the equipment used in this study is shown in Figure 1. The eyeglasses are embedded with an IMU on the right temple. The IMU system (LSM6DS3-TR; 2.5 × 3 × 0.83 mm) includes a 3D accelerometer (range: ±2 g; sensitivity: 0.061 mg/LSB; noise: 90 μg/√Hz) and 3D gyroscope (range: 250°/s; sensitivity: 0.088 m°/s/LSB; noise: 5 m°/s/√Hz) was set at a sampling frequency of 26 Hz. A retroreflective marker was positioned close to the IMU (on the right temple of the smart eyeglasses; Figure 2). For both systems, the vertical axis represented the gravitational axis while participants were standing. An additional retroreflective marker was placed on the participant’s right index finger. This marker was used to synchronize both systems in order to acquire simultaneous data. Participants had to tap the right arm of the eyeglasses with their right index finger. This produced a specific detectable pattern that was easily identified in the optoelectronic signal by measuring the distance between the two retroreflective markers. The accelerometer of the IMU recorded a separate detectable pattern when the eyeglasses were tapped. The synchronization was then performed offline by determining the exact timestamps of the two measurements of this event. For all analyses, the optoelectronic and IMU signals were synchronized by aligning the two timestamps of this event.

The vertical acceleration measurements recorded by the IMU of the eyeglasses were sent to a smartphone application (Driver by Ellcie-Healthy, Antibes, France, Version 8.8) through a Bluetooth connection. The OptiTrack motive software (Naturalpoint, Inc., Corvallis, OR, USA, Version 1.10) was used to record the retroreflective marker position. To obtain the vertical acceleration of the retroreflective marker, the marker position was derived twice. All data recorded with both systems was processed with a MATLAB program (The MathWorks, Inc., Natick, MA, USA, version R2018a). The acquisition frequencies were 26 Hz and 52 Hz for the eyeglasses and the OptiTrack, respectively. 

### 2.4. Analysis

**Figure 3:**
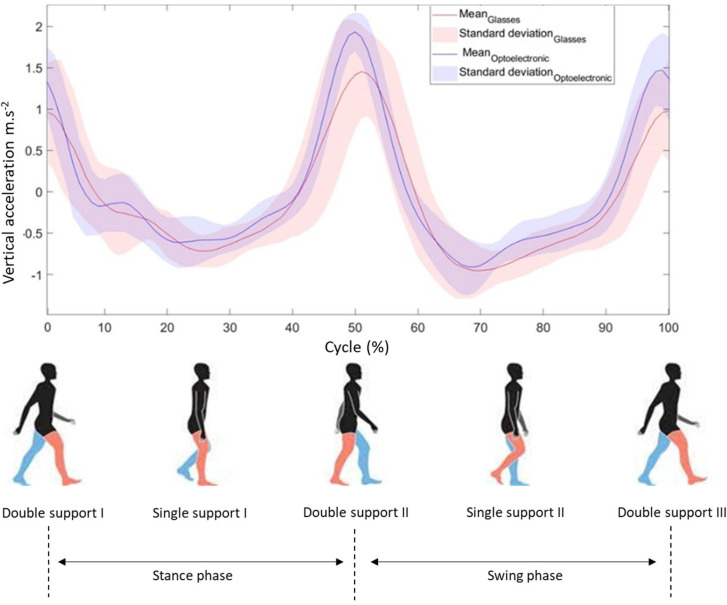
Acceleration signals from the glasses and optoelectronic system during gait cycle in 0.90 m/s speed without a cervical collar for one participant (red and blue colors indicate the right and the left lower limb, respectively).

For both recorded vertical accelerations (i.e., eyeglasses and optoelectronic system), a 6th order Butterworth filter was applied to suppress the noise. Filtered vertical accelerations were interpolated to obtain the same number (i.e., 100) of points for both signals (i.e., eyeglasses and optoelectronic) and for each gait cycle. From these signals, the different gait steps were identified and a cross-correlation on both vertical accelerations was applied to synchronize both systems. Figure 3 displays a gait cycle of the data acquired and processed for the two systems (i.e., OptiTrack and eyeglasses). A gait cycle is composed of two steps, each of which is characterized by a peak of acceleration in the vertical axis. The signals obtained from the optoelectronic system and the IMU embedded in the eyeglasses were similar. Step duration and step length of the gait were determined from the peak acceleration of both systems. To calculate step duration, the time difference between consecutive peaks was used for each recorded step. For all experimental conditions, step length was obtained by multiplying step duration by walking speed. Step length was normalized by the lower limb length of each participant. Step duration and step length normalized by the lower limb length were compared between both systems for all steps recorded during all experimental conditions.

### 2.5. Statistics

Statistical software (StatSoft, Version 8.0, Tulsa, OK, USA) was used for statistical analysis. The normality of the data distribution was confirmed by the Shapiro–Wilk test. A two-factor ANOVA with repeated measures (i.e., with or without a cervical collar × intra- and inter-session) for each variable was realized in order to assess the systematic errors within the number of steps of each speed, respectively. The effect size was calculated from partial eta-squared values (η^2^*p*) and the significance level was set to *p* < 0.05. To identify the specific mean differences, a Bonferroni post hoc test was performed. Moreover, intraclass correlation coefficients (ICCs: 1–k [32]) with 95% confidence intervals were calculated to evaluate the relative reliability of the step duration and step length within each experimental condition. ICCs greater than 0.90 indicate an excellent reliability, ICCs between 0.75 to 0.90 represent good reliability. ICCs between 0.5 and 0.75 are considered as a moderate reliability, while ICCs below 0.5 reflect poor reliability [33]. We first calculated the average values of each parameter over the 30 s period of each experimental condition. To evaluate the data variability between the participants across each test session, a coefficient of variation (CV) was calculated for each variable. The acceptability threshold of this coefficient was set to 10% [34]. The absolute reliability index was calculated as the standard error of measurement (SEM) [35]. This value was not impacted by the inter-subject variability. An excellent absolute variability was achieved when the SEM values were less than 10% of the average test or retest values. The minimal detectable change values at the confidence level of 95% were calculated (MDC95). To assess construct and concurrent validity, we used the Pearson’s correlation coefficient (r) and Bland–Altman graph with 95% limits of agreement. The plots present comparisons between the measurements of step duration and step length using the two tools [36].

## 3. Results

### 3.1. Intra- and Inter-Session Reproducibility of Step Duration and Step Length Measured with the Glasses and Optoelectronic System

The mean values of step duration and step length normalized by the lower limb length are presented in Table 1 and Table 2, respectively, for both systems (i.e., glasses and OptiTrack) for all experimental conditions. 

For the IMU embedded in the glasses, no significant interaction (*p* > 0.05) was observed for step duration whatever the session and the speed considered: low, F(37,1406) = 1.24, η^2^*p* = 0.03; moderate, F(42,1596) = 0.95, η^2^*p* = 0.02 and high, F(43,1634) = 1.14, η^2^*p* = 0.03. Similarly, no significant interaction (*p* > 0.05) was observed for step length whatever the session and the speed considered: low, F(37,1406) = 1.24, η^2^*p* = 0.03; moderate, F(42,1596) = 0.93, η^2^*p* = 0.02 and high, F(43,1634) = 1.18, η^2^*p* = 0.03.

For the optoelectronic system, no significant interaction (*p* > 0.05) was noted during low speed (e.g., F(37,1406) = 0.76, η^2^*p* = 0.02 for both step duration and step length) and moderate speed (e.g., step duration, F(42,1596) = 0.71, η^2^*p* = 0.02 and step length, F(42,1596) = 0.71, η^2^*p* = 0.02). For the highest speed, a significant cervical collar × intra- and inter-session interaction was found for both the step duration (F(43,1634) = 1.45, *p* < 0.05, η^2^*p* = 0.04) and step length (F(43,1634) = 1.46, *p* < 0.05, η^2^*p* = 0.04). However, the Bonferroni post hoc analysis did not highlight any significant difference.

### 3.2. Intra- and Inter-Session Reliability of the Glasses and the Optoelectronic System

Intra-session values of ICC, SEM, MDC95 and CV of step duration and step length are, respectively presented in Table 1 and Table 2 for both the glasses and the optoelectronic system. 

For the glasses, the ICC values of step duration were higher than 0.936 for both sessions and for the three speeds, which indicates an excellent reliability. The ICC values of the data obtained with the optoelectronic system were higher than 0.981 for both sessions, representing an excellent reliability, too. All ICC values of the step length normalized by the lower limb length, recorded with the glasses, were higher than 0.955 for both sessions, indicating an excellent reliability. An excellent reliability was also observed for the optoelectronic system, with ICC values higher than 0.981 for both sessions. 

The SEM values of the step duration and step length (Table 1 and Table 2, respectively) were smaller than 10% of the average test value for both systems. Hence, an excellent absolute reliability of both systems was observed for both parameters (i.e., temporal and spatial) in all experimental conditions. 

The CV values were between 5.719% and 7.838% for the glasses for the step duration and step length normalized by the lower limb length for both testing sessions. For the optoelectronic system, the CV values were between 2.292 and 4.632%. All CV values were less than 10%, indicating a low variability. 

Inter-session values of ICC, SEM and MDC95 of the step duration and step length recorded by the glasses and optoelectronic system are displayed in Table 3. For both parameters, an excellent absolute reliability of both systems was found, with SEM values less than 10% of the average retest value for both systems.

### 3.3. Concurrent Validity of Intra- and Inter-Session Reliability of the Glasses and the Optoelectronic System

**Figure 4:**
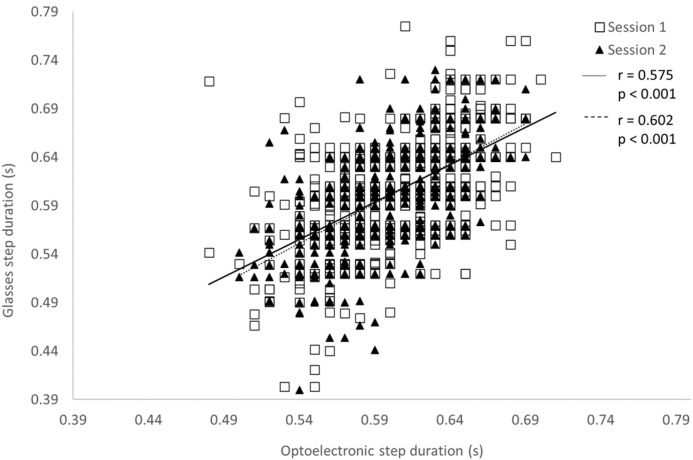
Pearson’s correlation of the step duration measured with the optoelectronic system and the glasses during the 30 s of gait at 0.90 m/s without a cervical collar for both test sessions.

**Figure 5:**
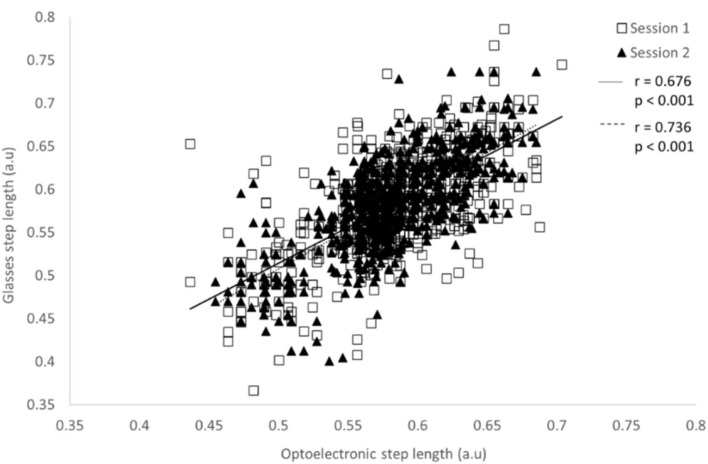
Pearson’s correlation of the step length normalized by the lower limb length measured with the optoelectronic system and the glasses during 30 s of gait at 0.90 m/s without a cervical collar for both test sessions.

**Figure 6:**
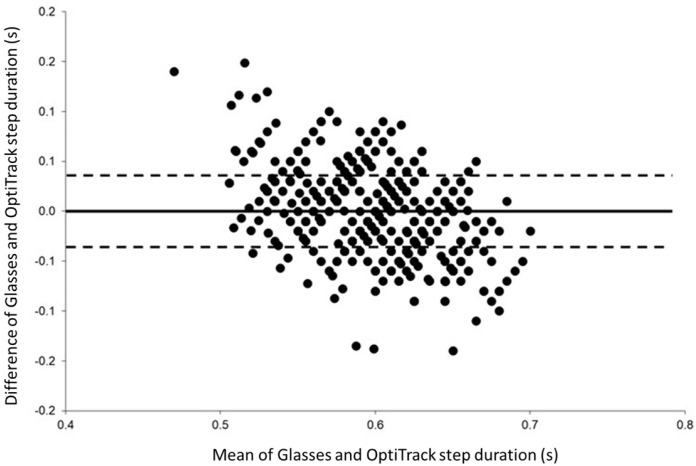
Bland-Altman plots of the step duration recorded without a cervical collar during the moderate speed condition (i.e., 0.90 m/s) for both testing sessions showing the differences between the glasses and OptiTrack against the mean of the two systems. The solid horizontal line represents the estimated bias, and the dashed lines represent the 95% limits of agreement.

The concurrent validity between the step duration measured with the glasses and the OptiTrack system is presented in Table 4. For all experimental conditions, significant Pearson’s correlations were observed (*p* < 0.001) for both systems and for both parameters. These coefficients were between 0.326 and 0.715 for the step duration, and between 0.364 and 0.736 for the step length. Figure 4 and Figure 5 present the correlations between the two systems for the step duration and step length, respectively, at the moderate speed (0.90 m/s) without a cervical collar. Bland–Altman plots were drawn for all experimental conditions. Figure 6 displays an example of Bland–Altman plots for the step duration during moderate speed without a cervical collar. 

## 4. Discussion

In this original study, a retroreflective marker was placed close to an IMU embedded in the right temple of smart eyeglasses to measure two major spatiotemporal parameters of gait. The step duration and step length were computed from the maximum vertical acceleration recorded at three different walking speeds and for two conditions of head stabilization (i.e., with or without a cervical collar) (Table 1 and Table 2). The reproducibility and reliability between the measurements were excellent for both systems whatever the experimental condition considered. In addition, excellent absolute reliability was observed for the step duration and step length recorded with the glasses. Lastly, the concurrent validity of the smart eyeglasses against the optoelectronic system was verified. 

In healthy adults, the values of the step duration and step length recorded with both the glasses and the optoelectronic system were in agreement with the values previously observed in the literature when measured with wearable sensors [37,38,39]. For example, our step duration values observed on the treadmill at the highest speed were similar to those previously measured with an accelerometer located at the lumbar level in young adults during level walking at a self-selected pace of ~1.4 m/s [39]. Whatever the measurement system (i.e., glasses or OptiTrack), the step duration and step length assessed were reproducible for both the intra- and inter-session for all experimental speed conditions (i.e., low, moderate and high speed). More importantly, no difference was observed between trials realized with and without a cervical collar. Consequently, measuring the step duration and step length during gait without head stabilization is feasible and reproducible with an IMU embedded in the glasses.

In addition to the reproducibility, the relative reliability of the glasses to measure the step duration (Table 1) and step length (Table 2) was excellent for all experimental conditions and both testing sessions. Indeed, the ICC values were between 0.936 and 0.993 for the step duration and between 0.955 and 0.987 for the step length. Moreover, the coefficient of variation was less than 8% for the glasses and less than 5% for the kinematic data, whatever the experimental condition. The SEM of the glasses was less than 10% of the mean for all speed conditions and for the measures realized with and without a cervical collar, representing an excellent absolute reliability [35], both intra- and inter-session. The SEM and absolute reliability of the optoelectronic system were similar to the values obtained with the glasses. Taken together, all these observations indicate that the measurement of step duration and step length is highly reliable and reproducible. Although these results could in part be ascribable to the constant speed imposed by the treadmill, our study also demonstrates that the vertical acceleration measured by the IMU embedded in the glasses can be used to determine step duration and step length.

Depending on the walking speed, the test–retest reliability of the spatiotemporal parameters between the two testing sessions was poor-to-excellent for the eyeglasses (0.311 < ICC < 0.923; Table 3) and moderate-to-excellent for the optoelectronic system (0.573 < ICC < 0.970; Table 3). Although, the relative reliability differed according to the speed, the SEM was less than 10%, indicating excellent absolute reliability for both the glasses and the optoelectronic system. The test–retest ICC values found for the glasses were comparable to or less than the values measured with other wearable sensors during level walking at self-selected speed [26,38]. Although the participants were familiarized with the experimental conditions, one possible reason could be related to the use of a treadmill and/or to the low speed influencing the gait patterns. Nevertheless, excellent absolute reliability was observed, suggesting that the glasses could be used to assess step duration and step length of gait in ecological situations.

The concurrent validity of the IMU embedded in the glasses and the optoelectronic system was determined for all experimental conditions and for both testing sessions. Significant weak-to-strong correlations were found between systems for all speeds during both conditions of head stabilization. A better correlation was generally observed for the moderate speed (i.e., 0.90 m/s) without a cervical collar (Table 4) for both the step duration and step length. Although in agreement with previous studies [37,40,41,42], which placed the accelerometer around the waist of the participants, we observed slightly lower correlation values than these studies. Bland–Altman plots were drawn for each experimental condition, and no systematic bias was present between the two systems. Consequently, both the glasses and the optoelectronic system are comparable to measure step duration and step length during gait assessment at different speeds.

This study has some limitations. First, only healthy and young participants were included in the present study. This was a necessary first step to validate the suitability of the glasses for the assessment of the step duration and step length before their application to older participants. Second, in our study, we adapted the threshold of the peak of each gait acquisition to prevent the incorrect identification of steps [42]. Third, gait analysis was realized in a laboratory setting and, in particular, the participants had to walk on a treadmill with an imposed constant speed. This experimental condition is far from daily life locomotion. The gait step length was obtained from the constant imposed speed of the treadmill, but in an ecological situation, gait speed is not known, unless a GPS (Global Positioning System) is integrated in the glasses. Another approach could use the inverted pendulum model [43] to extract spatiotemporal parameters from the acceleration signal [44,45,46]. Associated with the implementation of algorithms based on the vertical acceleration signal recorded from an IMU [45,46,47], the step length can be estimated and the eyeglasses could be used in ecological conditions. In this context, the daily life walking of elderly people could be monitored and, with the help of machine learning techniques, the variability of spatiotemporal parameters, providing an indicator of the risk of falling [48], could be assessed as well. Thus, elderly people wearing smart eyeglasses could be screened during daily life for risk of falling purposes and feel safer since this device already includes an alert application in case of falling.

A strength of the study was that the use of the glasses, an object of everyday life for most of the population, allowed step recognition from the acceleration measured on the vertical axis, whatever the face morphology of the participant and even if the glasses were not perfectly adjusted.

## 5. Conclusions

Gait analysis with wearable sensors has recently gained prominence, in both ecological and clinical conditions. The aim of this study was to validate an IMU embedded in the branch of eyeglasses to analyze gait in healthy participants. Overall, our results indicate that step duration and step length measured with the glasses are reproducible and valid. In future studies, step duration and step length will be investigated in both clinical and ecological settings and specifically in older populations to determine the reference values as measured by the eyeglasses. Given that the vertical acceleration recorded from an accelerometer positioned at head level can be used to (i) discriminate fallers and non-fallers through the recording of spatiotemporal parameters [7] and (ii) compute the variability of spatiotemporal parameters through machine learning techniques [41,46], the use of our eyeglasses seems to be a promising “non-intrusive” real life device to detect falls and/or to identify a potential decline in gait ability.

## Figures and Tables

**Table 1:** Mean step duration values, intraclass correlation coefficient (95% confidence interval), standard error of the measurement, minimum detectable change and coefficient of variation (%) measured with the optoelectronic system (OptiTrack) and the eyeglasses during 30 s of gait for both sessions.

	Mean Step Duration (Standard Error) in s		Intraclass Correlation Coefficient (95% Confidence Interval)		Standard Error of the Measurement		Minimum Detectable Change		Coefficient of Variation (%)	
	Session 1	Session 2	Session 1	Session 2	Session 1	Session 2	Session 1	Session 2	Session 1	Session 2
Eyeglasses										
0.72 WCC	0.684 (0.048)	0.676 (0.041)	0.975 (0.948–0.986)	0.970 (0.976–0.994)	**0.008**	**0.028**	0.023	0.013	7.034	6.118
0.72 CC	0.681 (0.049)	0.682 (0.047)	0.955 (0.974–0.993)	0.987 (0.969–0.992)	**0.008**	**0.010**	0.022	0.005	7.218	6.987
0.90 WCC	0.605 (0.037)	0.601 (0.034)	0.979 (0.940–0.984)	0.959 (0.945–0.985)	**0.005**	**0.007**	0.015	0.019	6.179	5.719
0.90 CC	0.587 (0.040)	0.593 (0.038)	0.979 (0.963–0.990)	0.993 (0.929–0.981)	**0.006**	**0.003**	0.016	0.009	6.754	6.402
1.1 WCC	0.557 (0.044)	0.552 (0.034)	0.949 (0.911–0.976)	0.950 (0.912–0.977)	**0.010**	**0.025**	0.028	0.021	7.838	6.101
1.1 CC	0.554 (0.042)	0.544 (0.036)	0.975 (0.956–0.988)	0.936 (0.888–0.970)	**0.007**	**0.009**	0.018	0.008	7.539	6.568
OptiTrack										
0.72 WCC	0.689 (0.030)	0.681 (0.031)	0.981 (0.966–0.991)	0.991 (0.984–0.996)	**0.004**	**0.011**	0.012	0.008	4.373	4.456
0.72 CC	0.688 (0.032)	0.684 (0.030)	0.985 (0.956–0.988)	0.982 (0.921–0.979)	**0.004**	**0.004**	0.011	0.003	4.632	4.380
0.90 WCC	0.605 (0.016)	0.601 (0.016)	0.994 (0.989–0.997)	0.993 (0.988–0.997)	**0.001**	**0.001**	0.004	0.004	2.726	2.654
0.90 CC	0.588 (0.018)	0.594 (0.016)	0.995 (0.990–0.997)	0.993 (0.988–0.997)	**0.001**	**0.001**	0.004	0.004	3.214	2.731
1.1 WCC	0.555 (0.015)	0.553 (0.013)	0.987 (0.978–0.994)	0.992 (0.986–0.996)	**0.002**	**0.001**	0.005	0.003	2.709	2.292
1.1 CC	0.547 (0.013)	0.545 (0.013)	0.994 (0.990–0.997)	0.992 (0.986–0.996)	**0.001**	**0.001**	0.003	0.003	2.486	2.309

Abbreviations: WCC, without a cervical collar; CC, with a cervical collar; and 0.72, 0.90 and 1.1 represent the treadmill speeds in m/s. Values in bold represent the SEM values less than 10% of the average test value.

**Table 2:** Mean step length normalized by the lower limb length, intraclass correlation coefficient (95% confidence interval), standard error of the measurement, minimum detectable change and coefficient of variation (%) measured with the optoelectronic system (OptiTrack) and the eyeglasses during 30 s of gait for both sessions.

	Mean Step Length (Standard Error) (a.u)		Intraclass Correlation Coefficient (95% Confidence Interval)		Standard Error of the Measurement		Minimum Detectable Change		Coefficient of Variation (%)	
	Session 1	Session 2	Session 1	Session 2	Session 1	Session 2	Session 1	Session 2	Session 1	Session 2
Eyeglasses										
0.72 WCC	0.541 (0.038)	0.535 (0.033)	0.970 (0.948–0.986)	0.987 (0.976–0.994)	**0.007**	**0.004**	0.018	0.011	7.034	6.118
0.72 CC	0.539 (0.039)	0.540 (0.037)	0.975 (0.956–0.988)	0.955 (0.921–0.979)	**0.006**	**0.008**	0.017	0.022	7.218	6.987
0.90 WCC	0.585 (0.036)	0.581 (0.033)	0.979 (0.964–0.990)	0.984 (0.972–0.993)	**0.005**	**0.004**	0.014	0.012	6.179	5.719
0.90 CC	0.567 (0.038)	0.574 (0.037)	0.983 (0.970–0.992)	0.977 (0.961–0.990)	**0.005**	**0.006**	0.014	0.015	6.754	6.402
1.1 WCC	0.659 (0.053)	0.652 (0.040)	0.972 (0.951–0.987)	0.975 (0.956–0.988)	**0.009**	**0.006**	0.009	0.006	7.838	6.101
1.1 CC	0.656 (0.049)	0.643 (0.042)	0.985 (0.974–0.993)	0.968 (0.944–0.985)	**0.006**	**0.008**	0.006	0.008	7.539	6.568
OptiTrack										
0.72 WCC	0.545 (0.024)	0.539 (0.024)	0.981 (0.966–0.991)	0.991 (0.984–0.996)	**0.003**	**0.002**	0.009	0.007	4.373	4.456
0.72 CC	0.545 (0.025)	0.540 (0.037)	0.985 (0.974–0.993)	0.982 (0.969–0.992)	**0.003**	**0.003**	0.008	0.009	4.632	4.380
0.90 WCC	0.585 (0.016)	0.581 (0.015)	0.996 (0.993–0.998)	0.996 (0.994–0.998)	**0.001**	**0.001**	0.003	0.003	2.726	2.654
0.90 CC	0.569 (0.018)	0.575 (0.016)	0.996 (0.993–0.998)	0.996 (0.993–0.998)	**0.001**	**0.001**	0.003	0.003	3.214	2.731
1.1 WCC	0.655 (0.018)	0.654 (0.015)	0.993 (0.988–0.997)	0.997 (0.994–0.999)	**0.001**	**0.001**	0.004	0.002	2.709	2.292
1.1 CC	0.647 (0.016)	0.644 (0.015)	0.997 (0.994–0.998)	0.996 (0.993–0.998)	**0.001**	**0.001**	0.003	0.003	2.486	2.309

Abbreviations: WCC, without a cervical collar; CC, with a cervical collar; and 0.72, 0.90 and 1.1 represent the treadmill speeds in m/s. Values in bold represent the SEM values less than 10% of the average test value.

**Table 3:** Test–retest of the spatiotemporal parameters of the eyeglasses and OptiTrack system without a cervical collar for the three tested speeds. A: step duration and B: step length normalized by the lower limb length.

**(A)**			
**Step Duration**	**Intraclass Correlation Coefficient** **(95% Confidence Interval)**	**Standard Error of the Measurement**	**Minimum Detectable Change**
Eyeglasses			
0.72	0.311 (0.174–0.522)	**0.023**	0.063
0.90	0.854 (0.669–0.940)	**0.009**	0.024
1.1	0.654 (0.297–0.851)	**0.014**	0.038
OptiTrack			
0.72	0.573 (0.186–0.806)	**0.017**	0.047
0.90	0.884 (0.730–0.952)	**0.004**	0.010
1.1	0.934 (0.841–0.973)	**0.005**	0.015
**(B)**			
**Step Length**	**Intraclass Correlation Coefficient (95% Confidence Interval)**	**Standard Error of the Measurement**	**Minimum Detectable Change**
Eyeglasses			
0.72	0.570 (0.410–0.749)	**0.018**	0.049
0.90	0.923 (0.816–0.969)	**0.006**	0.017
1.1	0.484 (0.065–0.758)	**0.018**	0.049
OptiTrack			
0.72	0.573 (0.186–0.806)	**0.013**	0.037
0.90	0.937 (0.849–0.975)	**0.003**	0.007
1.1	0.970 (0.926–0.739)	**0.002**	0.007

Abbreviations: 0.72, 0.90 and 1.1 represent the treadmill speed in m/s. Values in bold represent the SEM values less than 10% of the average retest value.

**Table 4:** Concurrent validity of the spatiotemporal parameters between the glasses and the optoelectronic system.

	Pearson’s Correlation (r)			Bland–Altman		
	Session 1	Session 2	Global	Bias (Mean Difference)	Lower Limit	Upper Limit
Step Duration						
0.72 WCC	0.368 ***	0.715 ***	0.563 ***	0.005	−0.115	0.124
0.72 CC	0.556 ***	0.538 ***	0.545 ***	0.005	−0.111	0.121
0.90 WCC	0.575 ***	0.602 ***	0.588 ***	0.000	−0.079	0.078
0.90 CC	0.452 ***	0.587 ***	0.503 ***	0.001	−0.099	0.101
1.1 WCC	0.326 ***	0.503 ***	0.393 **	0.000	−0.095	0.095
1.1 CC	0.483 ***	0.496 ***	0.488 **	−0.004	−0.095	0.088
Step Length *						
0.72 WCC	0.364 ***	0.714 ***	0.563 ***	0.004	−0.091	0.094
0.72 CC	0.554 ***	0.535 ***	0.545 ***	0.004	−0.088	0.096
0.90 WCC	0.676 ***	0.736 ***	0.706 ***	0.000	−0.076	0.075
0.90 CC	0.681 ***	0.699 ***	0.689 ***	0.001	−0.084	0.086
1.1 WCC	0.509 ***	0.654 ***	0.560 ***	−0.001	−0.116	0.114
1.1 CC	0.632 ***	0.628 ***	0.628 ***	−0.004	0.106	−0.115

Abbreviations: WCC, without a cervical collar; CC, with a cervical collar; and 0.72, 0.90 and 1.1 represent the treadmill speeds in m/s. *, Normalized by the lower limb length. ** *p*-values correlation less than 0.05. ***, *p*-values correlation less than 0.001.

## Data Availability

The data that support the findings of this study are available on request to the authors.
